# The Tell me tool: The development and feasibility of a tool for person‐centred infertility care

**DOI:** 10.1111/hex.13455

**Published:** 2022-02-26

**Authors:** Eva W. Verkerk, Ester A. Rake, Didi D. M. Braat, Willianne L. D. M. Nelen, Johanna W. M. Aarts, Jan A. M. Kremer

**Affiliations:** ^1^ Department of IQ Healthcare, Radboud University Medical Center Radboud Institute for Health Sciences Nijmegen The Netherlands; ^2^ Knowledge Institute of Medical Specialists Utrecht The Netherlands; ^3^ Department of Obstetrics and Gynecology Radboud University Medical Center Nijmegen the Netherlands; ^4^ Department of Obstetrics and Gynecology Amsterdam UMC Amsterdam The Netherlands

**Keywords:** infertility, patient‐centred care, person‐centred care, user‐centred design

## Abstract

**Background:**

An important—and often missing—element of person‐centred care is the inclusion of individual patients' values and preferences. This is challenging but especially important for high‐burden fertility treatments. We describe the development of a clinical tool that aims to facilitate the delivery of person‐centred fertility care by giving insight into the patients' values and preferences.

**Methods:**

We developed the Tell me tool following the three principles of user‐centred design: (1) early and continual focus on users; (2) iterative design; (3) measurement of user behaviour. Accordingly, our methods consisted of three phases: (1) conducting semi‐structured interviews with 18 couples undergoing fertility treatment, followed by a consensus meeting with relevant stakeholders; (2) performing seven iterative improvement rounds; (3) testing the feasibility of the tool in 10 couples.

**Results:**

The Tell me tool consists of a ranking assignment of 13 themes and two open‐ended questions. These themes relate to the couples' wellbeing and experience of the treatment, such as mental health and shared decision making. The open‐ended questions ask them to write down what matters most to them. The field test showed variation between the individual patients' answers. The tool proved to highlight what is important to the individual patient and gives insight into patients' personal contexts.

**Conclusions:**

We developed a tool that gives insight into the values and preferences of the individual patient. The tool seems feasible for facilitating person‐centred fertility care.

**Patient or Public Contribution:**

The tool was developed with a user‐centred design that strongly involved patients.

## 1 INTRODUCTION

In the last decades, we have seen a growing trend toward person‐centred healthcare.^1^ Person‐centred care has several advantages. It can improve health outcomes, patient's wellbeing, the patient–clinician relationship, patients' experiences with care and it can lower medical costs.^2^, ^3^, ^4^, ^5^, ^6^, ^7^, ^8^, ^9^

Patients with a chronic disease or a condition associated with a heavy burden of treatment can especially benefit from person‐centred care.^10^ A burdensome treatment, both physically and emotionally, is in vitro fertilization with intracytoplasmic sperm injection (ICSI), for which semen needs to be surgically retrieved through percutaneous epididymal sperm aspiration (PESA) or testicular sperm extraction (TESE). This treatment involves both partners, includes frequent and often emotional visits to the clinic and requires close, multidisciplinary collaboration.

Current literature defines several themes that are important to infertile patients, such as effectiveness, time and genetic parentage.^11^, ^12^ However, these studies take the approach of patient‐centred care. Although many similarities exist between person‐ and patient‐centred care (both include aspects such as empathy, shared decision‐making, communication and relationships) the main difference is that patient‐centred care aims for a functional life while person‐centred care is broader and aims for a meaningful life.^1^ Patients receiving infertility treatment might benefit from a person‐centred approach because the outcome of this treatment and possibly stopping the treatment relate to their meaningfulness of life.^13^ Living a life without biological children or without children at all might force couples to redefine meaning in life.

Despite many initiatives, person‐centred care remains hard to accomplish because many elements are involved, such as communication between patients and clinicians, coordinated care and the individualized context of the patient.^1^ One component is adequate incorporation of patients' preferences into healthcare decisions.^14^ However, this appears to be a tough nut to crack. Many clinicians believe that they already integrate patient preferences into their recommendations,^15^, ^16^ and as Mulley et al.^15^ put it: ‘a preference misdiagnosis generally goes unnoticed’.

Misdiagnosis of preferences impedes person‐centred care delivery. As communication is a dyadic concept involving efforts from both the patient and the physician,^17^ strategies targeted at both groups could help to improve person‐centred care. First, the clinician needs to get acquainted with the patient and his/her preferences.^18^ Second, patients should be able to voice their values and preferences. However, it is not easy for patients to engage in their care.^19^ Patients need to be empowered to participate in the consultation;^20^, ^21^ moreover, they need time to construct an informed preference. Preparation in formulating preferences in advance could encourage patient engagement during the consultation.^21^, ^22^

A tool could facilitate both patients and clinicians in: (1) supporting the patient to form and voice their preferences and (2) supporting the clinician to correctly estimate these preferences to deliver person‐centred care. Tools that facilitate infertile patients to think and speak up about what is important to them in their fertility treatment and life are particularly promising because studies have shown that clinicians underestimate the importance of person‐centred care and tend to assume that the couple's only goal is to become pregnant.^16^, ^23^ A recent review showed that interventions for patients, their families and practitioners could improve patient‐centredness of care.^24^ For example, a tool that was developed for hospitalized patients showed a positive effect on patient participation.^25^, ^26^ A review focusing on shared decision‐making, which is an aspect of person‐centred care, concluded that patients who use decision aids feel clearer about their values.^27^

An existing theoretical framework defines several themes that are important to infertile patients,^12^ but this framework does not provide an intervention that can be used in clinical practice and does not use a person‐centred viewpoint. To our knowledge, no tools exist for facilitating a person‐centred approach for both patients and clinicians in complex fertility treatment. Therefore, we aimed to develop a tool, the ‘Tell me tool’ for couples in need of PESA/TESE‐ICSI treatment, that clarifies individual patients' values and preferences, facilitates patients to voice these and clinicians to understand them. This study describes the development of a person‐centred tool, using an iterative design and evaluates the usability of the tool in practice.

## 2 METHODS

### 2.1 Study design

We aimed to develop an instrument that would support patients in forming and voicing their preferences and clinicians in correctly understanding these preferences to deliver person‐centred care. We were inspired by the literature on goal setting and patient empowerment^28^, ^29^, ^30^, ^31^, ^32^ and wanted the tool to help patients construct their preferences by providing a list of ‘goals’ that could be important to them. These goals could then be discussed with their clinician.

**Figure 1:**
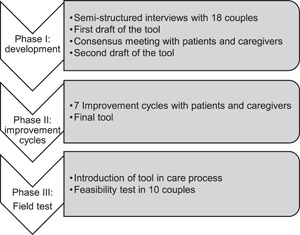
Overview of the methods used in this study

We developed the tool using a user‐centred development approach that includes feedback from both patients and clinicians as the most important stakeholders.^33^ A user‐centred design was originally used for the development of interactive software technologies in fields outside healthcare.^34^ Yet such a concept could also facilitate the design of healthcare interventions.^33^, ^35^ We used this approach following the three principles of user‐centred design: (1) early and continual focus on target users; (2) measurement of user behaviour; (3) iterative design.^36^, ^37^ In our setting, patients and clinicians were the target users of the intended tool. For that reason, they were involved from the very beginning of the development process. Accordingly, our methods consisted of three phases: (1) developing a draft of the tool; (2) improving the tool in iterative improvement cycles; (3) testing the usability of the tool. Figure 1 gives an overview of the methods used.

The Research Ethics Committee of the Radboud University Medical Center judged that ethical approval was not required under Dutch National Law. The study was performed in line with the principles of the Declaration of Helsinki. This study was reported using the Consolidated criteria for reporting qualitative studies (COREQ, see Appendix SA).

### 2.2 Setting

This study focused on infertile couples of which the man had been diagnosed with azoospermia, which required a PESA/TESE‐ICSI treatment. Because this is the most burdensome of fertility treatments and involves treating both the man and the woman, discussing person‐centred care aspects might be very important for these patients. This intensive treatment starts with surgically obtaining semen from the man, subsequently hyperstimulating the woman's ovaries with hormones, harvesting oocytes, fertilizing them by injecting a sperm cell and transferring the embryos to the woman's uterus. Dutch basic healthcare insurance covers three PESA/TESE‐ICSI treatment cycles for women up to 43 years old. The tool development took place at the Department of Obstetrics and Gynecology of the Radboud University Medical Centre in the Netherlands in 2014 and 2015.

### 2.3 Phase I: Development of the tool

As the Tell me tool aimed to facilitate both patients and their clinicians in their conversations, both stakeholder groups were involved in its development. This was in line with the first principle of user‐centred development: early and continual focus on target users.

E. V. performed face‐to‐face, semi‐structured interviews with couples that were undergoing or were eligible for PESA/TESE‐ICSI, to identify themes that were important to them. We invited couples from different stages in the care process and with different ages. The topic guide was developed based on literature related to the patient's experience of the treatment process and the patient's wellbeing during and after infertility treatment (see Supporting Information 1)^10^, ^38^, ^39^, ^40^, ^41^, ^42^, ^43^ leading to three main focus points. First, the interviewer asked the couples open‐ended questions on what mattered to them in their fertility care. Second, the interviewer summed up the identified themes from the literature and inquired whether the couple had any additional items. Thirdly, the couple discussed and answered the themes that mattered most to them. The man and woman each gave a top three of most important themes; we assigned three points to each patients' most important theme, two points to the next important and one point to the third most important theme.

We interviewed until we achieved data saturation. For confirmation purposes, we conducted two more interviews. Interviews were audio‐recorded and transcribed verbatim. E. V. and E. S. independently coded the transcripts using an inductive thematic analysis in Atlas.ti.^44^ In this approach, the analysis was data‐driven and themes were constructed without a pre‐existing frame. Codes were compared, and when they differed, we discussed until we reached a consensus, if necessary with a third person (W. N.). All codes were grouped into categories derived from the data through constant comparison and review.

E. V., D. B., W. N. and J. K. created a first draft of the Tell me tool based on the input of the patient interviews and the concept of goal setting. The 10 themes that received the highest number of points in the interviews were included in the draft tool. This draft was presented in a consensus meeting with stakeholders who were involved in PESA/TESE‐ICSI care (one nurse, one secretary, one nurse practitioner, one patient, two fertility physicians, one urologist, the head of the fertility lab and the head of the fertility department). The patient was a representative of the Dutch patient association for fertility care and was experienced in participating in research. The set of themes, layout, distribution, implementation and name of the Tell me tool were discussed until consensus was achieved. With this information, we developed the second version of the Tell me tool.

### 2.4 Phase II: Iterative improvement cycles

**Figure 2:**
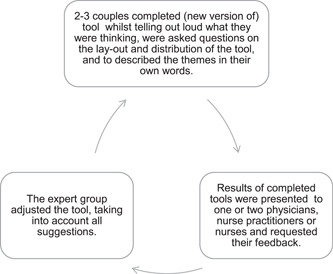
Method of iterative improvement cycles in Phase II of the development of the tool: cycles to adapt and adjust the tool, leading to new versions

Following the second and third principles of user‐centred development (i.e., measurement of user behaviour and iterative design), the Tell me tool went through several iterative improvement cycles. In these cycles, user experiences and approaches to fill in the tool were measured using the think‐aloud method.^45^ We asked couples to complete the tool while telling out loud what they were thinking. The completed tools and results were discussed with the physicians and subsequently, the expert team adapted the tool into a new version (Figure 2). We concluded the improvement cycles when no more considerable issues emerged.

### 2.5 Phase III: Field test

During Phase III, the tool was introduced into daily practice. For optimal implementation, we discussed the distribution, processing and use of the tool in the aforementioned consensus meeting and the improvement cycles. We held educational meetings with all clinicians involved in PESA/TESE‐ICSI care. Afterwards, we sent them an instruction form with a summary of the meeting.

We tested the usability of the tool from the patient's perspective in a field test. The main goal was to test whether the tool supports patients to form and voice their preferences. PESA/TESE‐ICSI couples that were referred to the Radboud university medical centre received the tool. We collected 20 completed tools from 10 couples, of which 10 were completed by women and 10 were completed by men. We evaluated patients' understanding of the tool, ability to complete the tool correctly and what values and preferences they wrote down. Subsequently, we calculated the mean ratings and scores per theme, as well as the number of times a theme received the highest score from a patient. We then thematically coded the patients' answers onto the open questions in Atlas.ti.

## 3 RESULTS

### 3.1 Phase I: Development of the tool

From the interviews with 18 PESA/TESE‐ICSI couples, we reached a consensus on 17 final themes (see the Supporting Information 2), with which we could develop the first draft of the tool. The most important themes differed slightly between men and women: men found being involved and comfort more important, while women found accessibility and continuity more important. For that reason, we decided to develop a version of the tool for men and one for women, each version containing a different set of themes. To describe the themes in a comprehensible way for patients, we used descriptions directly derived from the interviews. For example, ‘competence of staff’ was described as ‘trusting the clinicians’ expertize', and ‘accessibility’ as ‘being able to ask questions’. We left some room for patients to come up with their own topics. The first draft of the tool consisted of two parts. In the first part of the tool, patients were asked to rate all themes on importance and distribute 100 points among them. The second part of the tool contained open‐ended questions for additional in‐depth information on the values and preferences of the patient. The tool was developed as a booklet that patients could bring to the consultation and that the clinician could read and use during the appointment.

During the consensus meeting, the PESA/TESE‐ICSI‐related stakeholders voiced various remarks and ideas on the first draft of the tool as well as its use in clinical practice. Consensus on these points was however reached fairly quickly. Two examples of feedback: the stakeholders agreed that the tool should be easy to administer and it should be short, and the clinicians wished clarification on why patients found certain themes important, indicating that there should be space for the patient to explain. A second version of the tool was created using these recommendations (Supporting Information 3).

The clinicians preferred to read the tool before the consultation instead of receiving the tool from the patient during the consultation. Also, due to time constraints, both the clinician and the patient expected that only one or two themes could be discussed during the consultation. The remarks were taken into account for using the tool in daily practice (Phase III).

### 3.2 Phase II: Iterative improvement cycles

In total, 23 PESA/TESE‐ICSI couples and several nurses, nurse practitioners and physicians working at the Department of Obstetrics and Gynecology during this study were consulted in the improvement cycles. We adjusted the tool seven times until no more considerable issues emerged.

**Figure 3:**
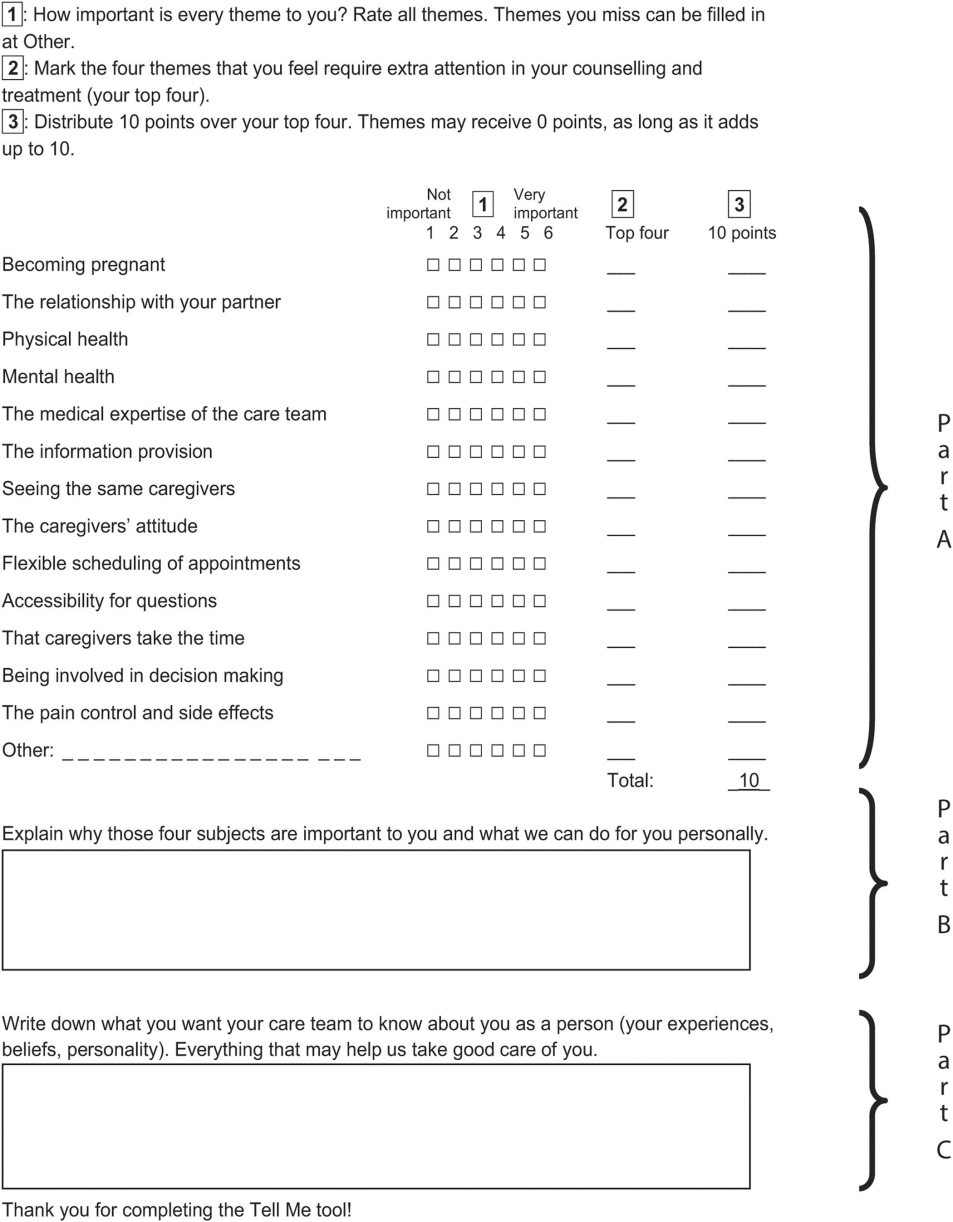
Final version of the tool, consisting of parts A, B and C. Part A involves three steps, accordingly the three columns in part A. Part B and C consist of open‐ended questions

Regarding the content of the tool, couples found it confusing that the tools for men and women sometimes differed in themes. We, therefore, decided to use the same themes for both genders. Some patients reported that what they feel is important is not always something they would like to talk about with their clinician. In addition, distributing 100 points proved to be quite hard. This led us to test different instructions, guided by what patients suggested, which resulted in the final instructions in part A of the tool (Figure 3) where respondents had to distribute 10 points. In addition, we added an open‐ended question inviting patients to explain their top four (part B, Figure 3). The two open‐ended questions from the first draft of the tool were merged into part C (Figure 3). Patients and clinicians found the open‐ended questions valuable because they gave room for personal information. Figure 4A,B shows the final Tell me tool, completed by the first couple of the field test.

Figure 4(A) First completed tool (final version) of the feasibility test (Phase III) of a couple: (A) women version and (B) men version

**Figure:**
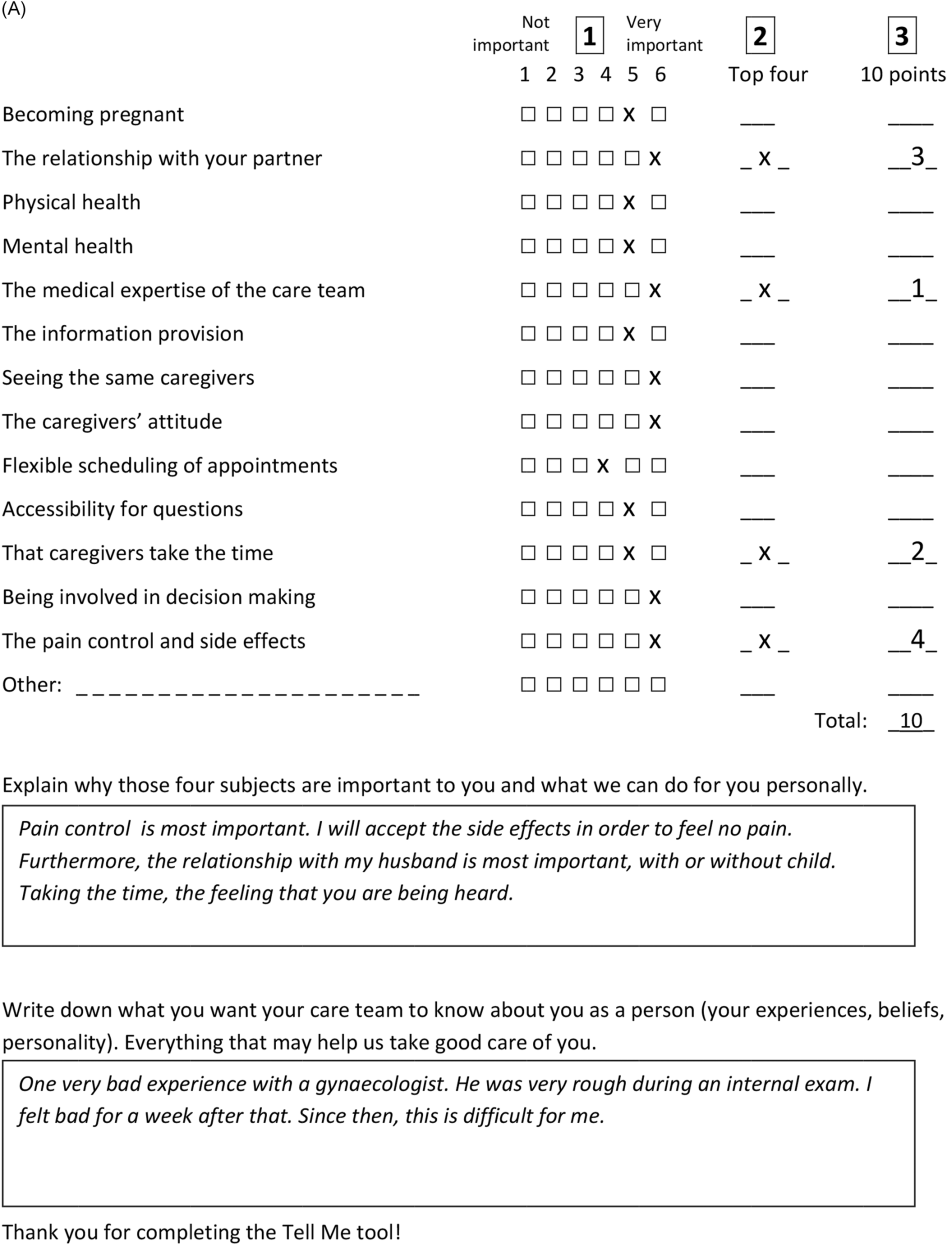

**Figure:**
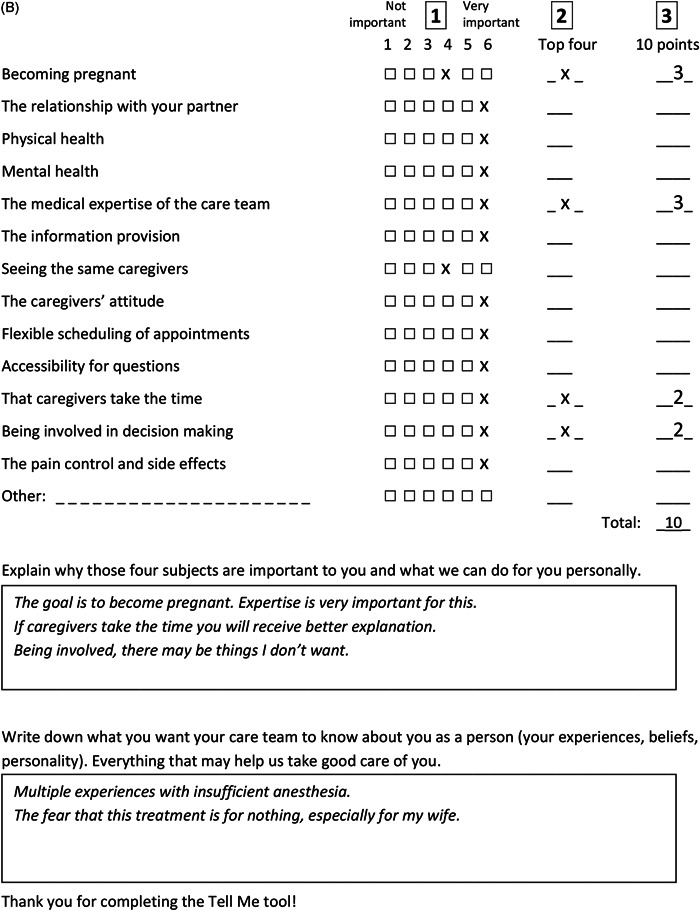

Regarding the clinical application of the tool, we designed the use of the tool in line with the feedback given in Phase I, for example, to embed the tool in already existing routines, thereby keeping the burden for patients and clinicians low.

### 3.3 Phase III: Field test

The field test aimed to give insight into the user experiences of patients. Based on the input from both the consensus meeting and the improvement cycles, the tool was used as follows: PESA/TESE‐ICSI patients, scheduled for intake, received the tool by mail together with their registration forms to ensure limited administrative time. This also met the patients' preference to complete the tool on paper. Next, patients returned the completed tool and the registration forms to the secretary, who added them to their medical records. In addition, E. V. wrote a summary of the patient's tool in the treatment overview. In doing so it would be easier to find for all clinicians, should they wish to read the summary before the consultation. Clinicians were instructed to discuss with the patients their most important themes during the consultation and where possible to personalize their care plan or counselling. Within 2 months, 10 couples completed and returned the tool. The women had a mean age of 29.2 years (SD = 3.6) and men a mean age of 29.7 years (SD = 3.4). Of the 20 men and women, 17 filled in the tool completely and answered at least one of the two open‐ended questions. One couple did not select a top four and did not distribute 10 points, but rated the themes and answered one of the open questions. Furthermore, one man did not answer the open‐ended questions. As the tool had been added to the patients' registration forms during a long time‐frame, we did not know how many patients received the tool and therefore could not calculate a response rate.

Both men and women rated the importance of all the themes highly, as can be seen in Table 1 (Step 1 of part A). The lowest mean was 4.1 (accessibility for questions) and the highest was 6 (relationship with partner). The distribution of 10 points over their top four themes showed more insight and variation between patients (Steps 2 and 3 of part A). Three out of nine women and two out of nine men did not include ‘becoming pregnant’ in their top four. Next to ‘becoming pregnant’, many other themes received the highest score. One man wrote down an extra theme: ‘determining cause and targeted approach’.

**Table 1:** Scores on importance, extra attention and top scores on themes in part A of the tool

	Step 1 mean importance score (min–max)^a^		Step 3 mean score for extra attention (min–max)^b^		Top score^c^	
Theme	Women (*n* = 10)	Men (*n* = 10)	Women (*n* = 9)	Men (*n* = 9)	Women (*n* = 9)	Men (*n* = 9)
Becoming pregnant	5.6 (4–6)	5.4 (4–6)	2.1 (0–4)	2.2 (0–4)	5	5
The relationship with your partner	6.0 (6–6)	6.0 (6–6)	2.2 (0–7)	2.1 (0–6)	3	4
Physical health	5.4 (5–6)	5.5 (4–6)	1.1 (0–3)	1.6 (0–5)	1	3
Mental health	5.6 (5–6)	5.6 (4–6)	0.6 (0–2)	0.6 (0–2.5)		1
Medical expertize of the care team	5.6 (4–6)	5.4 (3–6)	0.9 (0–3)	1.4 (0–3)	1	1
Information provision	4.8 (4–6)	5.2 (3–6)	0.4 (0–4)	0.1 (0–1)	1	
Seeing the same clinicians	5.0 (4–6)	4.7 (3–6)	0.0 (0–0)	0.0 (0–0)		
Clinicians' attitude	5.5 (4–6)	5.1 (4–6)	0.1 (0–1)	0.6 (0–5)		1
Flexible scheduling of appointments	4.7 (4–6)	4.6 (2–6)	0.3 (0–2)	0.0 (0–0)		
Accessibility for questions	4.7 (4–6)	4.1 (2–6)	0.2 (0–2)	0.2 (0–2)		
Clinicians taking the time	5.0 (4–6)	4.9 (4–6)	0.4 (0–2)	0.2 (0–2)		
Being involved in decision making	5.7 (4–6)	5.4 (4–6)	0.8 (0–3)	1.0 (0–3)	1	
Pain control and side effects	5.1 (4–6)	4.7 (2–6)	0.6 (0–4)	0.0 (0–0)	1	

^a^ Mean score on importance, where 1 = not important and 6 = very important (Step 1 in Figure 4A,B).

^b^ Mean score on what requires extra attention, 10 points were distributed over top four (Steps 2 and 3 in Figure 4A,B).

^c^ Number of times a theme received the highest score from a patient on requiring extra attention (Step 3 in Figure 4A,B); patients could distribute 10 points freely and some patients gave two items an equally highest score.

The majority of the patients' responses to the open‐ended questions concerned a further explanation of their top four assigned in part A. The most often mentioned themes were pregnancy, relationship, (physical) health and clinician's expertize. Seven out of the 10 women and four out of the 10 men commented on their wish to become pregnant. Most of the answers given explained why a certain theme was important, although some patients did not come up with a lot of explanation but merely repeated that a theme was important.

The relationship with my partner is most important. We have to go through this together. (Woman, age 32)

*We want to* become *pregnant, but our health should not suffer from this*. (Man, age 34)

*Information in* easy *language. Taking the time for a good explanation and for understanding the next steps*. (Woman, age 30)

Next to comments on their themes, several patients mentioned a personality trait and/or their (care) experiences from the past.

*I have an optimistic personality. In the past years I have conquered several setbacks and experienced that I can beat them on my own, which has made me stronger. Therefore, I believe that we* can *handle this treatment*. (Woman, age 30)

*Multiple* experiences *with insufficient anesthesia*. (Man, age 35)

Furthermore, several themes that were not directly attributable to one of the themes in the list were mentioned: that is, equal communication between patient and clinician, the honesty of the clinician, the feeling of being taken seriously, counselling for childlessness in case of failed treatment, and being dependent on others for pregnancy and physical health.

Figure 4A,B shows the answers of the first couple of the field test that completed the final tool to exemplify how the tool provides insight into an individual patient's preferences.

## 4 DISCUSSION AND CONCLUSION

### 4.1 Discussion

This paper describes the development and field test of the Tell me tool that aims to support both the patient and clinician to elicit a patient's values and preferences. During the user‐centred development process, patients and clinicians were involved in the design and optimization of the tool and its introduction into daily practice. The field test showed that patients were able to complete the tool, that the tool reflects variation and can discriminate between patients. Furthermore, the open questions helped explain why a certain theme was important for patients. These results suggest that the tool can give insight into the values and preferences of the individual patient. The elicitation of patients' preferences is an important step in the process of shared decision‐making,^46^ which is essential to delivering person‐centred care.^1^, ^31^ In general, it is known that clinicians' knowledge of patients' preferences enhances person‐centred care. Since the tool is developed to support patients and clinicians in communicating about patient's values and preferences and subsequently enable person‐centred care, we will discuss the potential of the tool through the concept of clinician–patient communication.

First, the tool was developed to support patients to formulate their values and preferences. The field test showed that it helped patients in forming their preferences. In Step 1 of part A, the tool showed large ceiling effects (Table 1), indicating that patients could not prioritize which topics were most important to them. After assigning a top four and writing down what mattered to them, a more focused overview of the patients' preferences emerged. Also, sending this tool to patients brings the message across that clinicians are genuinely interested in their patients' values and preferences. Therefore, the tool could not only help patients to form their preferences but also empower patients to voice them during the consultation. To our knowledge, there are no tools yet for preference elicitation in fertility care. Our tool could fill this gap and help infertile patients to voice their concerns and needs.

Second, this tool could facilitate the clinician in signalling the patients' preferences and starting the conversation on this topic, leading to more person‐centred care. With this study, we have not yet gained insight into whether clinicians deliver more person‐centred care when using the tool. Nevertheless, other similar tools have already shown this effect. For example, the Tell us card, developed for hospitalized patients,^26^, ^47^ contains two questions: ‘What is important for you today?’ and ‘What is important for you before you are discharged?’ The use of the Tell us card improved the patients' ability to participate in decisions. In another study in the United Kingdom, hospitalized patients received a ‘What matters to me?’ whiteboard on their bed.^48^, ^49^ Patients expressed goals and wishes, such as ‘having a good session with the physical therapist’ and ‘clear communication’. The board was well received by clinicians as well as patients. These studies indicate that tools that aim to support the clinician in getting to know what is important for the patient could be effective in delivering more person‐centred care.

Involving patients and clinicians in the development of the tool is part of the user‐centred design we applied in the methods. This technique has been used before in the development of preference elicitation tools. Other researchers in the Netherlands also involved end‐users in the development of their tool for long‐term care. This resulted in a web‐based tool that assists patients with preference elicitation during consultations with professionals.^50^

### 4.2 Comparison to the existing literature

Duthie et al.^12^ published a framework on treatment dimensions for patient‐centred fertility care based on patients' views. They reported ‘genetic parentage’ (genetic/biological connection to child) as one of the dimensions. This theme did not emerge in our interviews, which could be caused by our more specific population of PESA/TESE‐ICSI patients who are trying to become pregnant based on their own gametes. Furthermore, Duthie's framework incorporated financial costs of the treatment, while in our interviews patients rated this theme as least important. Therefore, this theme was not included in our tool. This could be explained by our different healthcare systems, with the Dutch healthcare system being more financially supportive compared to the United States healthcare system. The other four themes of Duthie's theoretical framework are similar to the themes in our tool for clinical practice. Since the development of our tool was based on a person‐centred approach, we also included additional themes that are not present in Duthie's patient‐centred framework. Especially, the last open‐ended question asking patients what is important for the clinician to know about their personal life is related to the concept of person‐centred care, focusing on a meaningful life. Furthermore, the topic ‘relationship with your partner’ is part of our tool, while it is not part of the existing patient‐centred care framework. Remarkable about this last topic was that maintaining a good relationship with your partner was ranked as the most important theme, while research shows that clinicians expected that becoming pregnant would be the most important theme for patients.^16^ Our results were in line with another study that assessed family‐building aspects in infertile couples. It was reported that a close and satisfying relationship with one's partner was the highest priority for couples.^51^

### 4.3 Strengths and limitations

The user‐centred design was the greatest strength of our study. We used this method for developing the content, layout and use of the tool. With the think‐aloud method in the improvement cycles, we improved the tool so that the majority of patients would be able to complete it. A second strength is that the tool is intended and feasible for both partners in the infertility care pathway, which is very unique. A limitation of the tool is that we could not include all themes that emerged in the patient interviews. We chose to omit the least important themes because users recommended that the tool should not exceed one page. However, since patients had the opportunity to add additional themes on the dotted line after ‘other’ and in their answers to the open questions, we are confident that there was enough opportunity for patients to indicate what mattered to them. A second limitation of our study was the involvement of patients during the consensus meeting (Phase I). Only one patient representative participated, and the first draft of the tool was developed by the research team. In our view, we compensated for this by involving many patients in the improvement cycles (Phase II) and in the field test (Phase III). A third limitation was the limited information on demographics of the 10 couples who completed the tool. We only collected their age; therefore, we cannot estimate whether our findings are transferable to all infertile patients.

### 4.4 Practice implications

The tool showed variation in scores and answers to open questions between patients. This variability can be used by clinicians to adjust their counselling to individual patients' needs, for example, a clinician would counsel a patient who allocates the most points to ‘being involved in decision‐making’ differently from a patient who allocates the most points to ‘pain control and side effects’. Our tool can support clinicians to correctly diagnose the patients' needs. However, this variation and the patients' ability to complete the tool need to be confirmed in a larger field test. From the patients' perspective, the obvious focus on pregnancy in fertility care can distract them from other aspects that are also important to them. The tool can encourage them to think about this and formulate their needs. This impact of our tool on the clinicians' delivery of care and the patients' care experience needs further study. The tool can also be promising in other healthcare settings in which the patient needs support to elicit their preferences and clinicians to diagnose them. The tool can be completed by both partners of a couple, which can be relevant for treatments that have a high impact and burden on both their lives. This can also be challenging when differing and even incompatible preferences emerge within the couples who then require the clinicians' time and guidance. However, discussing this at the start of the fertility treatment might help to increase the couples' mutual understanding and to set expectations of the treatment. Patients can be referred for psychological or couples' counselling if needed.

For future research, it is relevant to investigate quantitative differences in values and preferences between both partners in a couple. For example, in mixed‐sex couples who undergo infertility treatment, women respond in a different manner to this stressful period compared to men.^52^, ^53^ Accordingly, we hypothesize that men and women will complete the Tell me Tool differently. This should also be studied in same‐sex couples. Future research could also study whether and how the values and preferences of patients change over the course of the fertility treatment. Subsequently, it can be determined how often and at what moments the tool can be best used. At the start of the treatment and in‐between ICSI cycles would seem to be appropriate moments. The third direction for future research is the development of the tool for other settings and users. We expect that the tool can also be valuable for conditions that have a significant impact on a person's life and require a lot of care, such as other fertility treatments, chronic diseases or cancer. These patients especially can benefit from more person‐centred care. For use in other fertility treatments, we expect that the themes of the tool will also be relevant, although this would need to be verified. For use in other conditions, we recommend developing a new set of themes that are relevant for these patients.

Lastly, for the development of similar interventions, we recommend using a user‐centred design. Especially the iterative feedback cycles in which we were able to try out different versions of the tool (language, questions, explanation, lay‐out), have helped us improve the tool. These approaches could enhance delivering person‐centred care.

### 4.5 Conclusion

The Tell me tool appears to be feasible for facilitating the delivery of person‐centred fertility care since the tool gives a reflection of the patients' values and preferences, and there is variation between the individual patients' answers. The user‐centred design was an important element in developing the Tell me tool since the feedback of end‐users heavily influenced the content, layout and use of the tool. Future research is necessary to confirm the effect of the tool for person‐centeredness fertility care.

## CONFLICT OF INTERESTS

The authors declare that there are no conflict of interests.

## AUTHOR CONTRIBUTIONS

Eva W. Verkerk, Willianne L. D. M. Nelen, Jan A. M. Kremer and Didi D. M. Braat designed the study. Interviews were performed by Eva W. Verkerk and coded by Eva W. Verkerk and EMS. The consensus meeting was performed by Eva W. Verkerk, Willianne L. D. M. Nelen, Kathrin Fleischer and Kathleen D'Hauwers. The first draft, all subsequent versions of the tool and the field test were developed by Eva W. Verkerk, Willianne L. D. M. Nelen and Jan A. M. Kremer. The improvement rounds were performed by Eva W. Verkerk. Ester A. Rake, Johanna W. M. Aarts and Eva W. Verkerk interpreted the data, drafted the manuscript and revised it critically. All authors approve of the final version of the manuscript for submission.

## Supporting information

Supplementary Information

Supplementary Information

Supplementary Information

Supplementary Information

## Data Availability

Research data are not shared.
