# Supplementary material for: The Tell me tool: The development and feasibility of a tool for person‐centred infertility care
Source: Health Expect. 2022 Feb 26;25(3):1081–93. doi: 10.1111/hex.13455 (PMC9122469; doi:10.1111/hex.13455)
Supplement: Supplementary file 1 — Supplementary Information [file HEX-25--s004.docx]

Consolidated criteria for reporting qualitative studies (COREQ):

Supplement to: EW Verkerk, EA Rake, DDM Braat, WLDM Nelen, JWM Aarts, JAM Kremer. The Tell me tool: the development and feasibility of a tool for person-centered infertility care. The Patient.

| Domain 1: Research team and reflexivity |  |
| --- | --- |
| Personal Characteristics |  |
| 1. Interviewer | EV performed the interviews with couples for developing the tool in phase I and guided the interviews for the improvement cycles for phase II. |
| 2. Credentials | EV: MSc BSc |
| 3. Occupation | EV: PhD candidate |
| 4. Gender | Female |
| 5. Experience and training | EV was trained to interview and analyze qualitative data. |
| Relationship with participants |  |
| 6. Relationship established | EV had not met the couples before interviewing them for this study. |
| 7. Participant knowledge of the interviewer | The reasons for the study were described in the information letter with which couples were approached. |
| 8. Interviewer characteristics | EV introduced herself at the start of an interview. |
| Domain 2: study design |  |
| Theoretical framework |  |
| 9. Methodological orientation and Theory | We used an inductive thematic analysis, in which the analysis is data-driven and the themes are strongly linked to the data themselves. In our analysis, we were informed by the literature on patient-centered fertility care. |
| Participant selection |  |
| 10. Sampling | Couples that were undergoing or were eligible for PESA/TESE-ICSI with a scheduled appointment with their care provider were invited to participate in an interview. We used purposive sampling to select couples from different stages in the care process and with different ages. |
| 11. Method of approach | Selected couples were sent an invitation and information letter. Approximately a week before their scheduled appointment, they were called to answer their questions regarding the interview and schedule the interview if they were willing to participate. |
| 12. Sample size | We interviewed 18 couples for phase I and 23 couples for phase II. |
| 13. Non-participation | 7 couples declined to participate in an interview for phase I and 7 couples declined to participate in phase II. We have no information on the reasons for couples to decline participation. |
| Setting |  |
| 14. Setting of data collection | We conducted face-to-face interviews in the hospital. |
| 15. Presence of non-participants | Only the interviewer and the couple were present. |
| 16. Description of sample | Of the 18 couples that were interviewed in phase I, 4 were at the start of the PESA/TESE-ICSI trajectory, 11 were undergoing the PESA/TESE-ICSI treatment, and 3 had finished their treatment and were scheduled for an evaluation appointment (of which 2 couples were not pregnant and 1 couple was pregnant).  The females of the 18 couples were aged 26-41 years with a mean of 32.8 years. The males of the 18 couples were aged 29-50 years with a mean of 38.1 years.  Of the 23 couples that were interviewed in phase II, 13 were at the start of the PESA/TESE-ICSI trajectory, 9 were undergoing the treatment, and 1 had finished their treatment and were pregnant.  The females of these couples were aged 23-41 years with a mean of 31,9 years. The males of these couples were aged 28-48 years with a mean of 39,1 years. |
| Data collection |  |
| 17. Interview guide | The guide for phase I is translated to English and provided as a supplementary file. In phase II we used the Think aloud method. Couples were asked to complete the tool while telling out loud what they were thinking. |
| 18. Repeat interviews | We did not perform repeat interviews. |
| 19. Audio/visual recording | All interviews were audio-taped. |
| 20. Field notes | No field notes were made. |
| 21. Duration | The duration of the interviews in phase I was on average 28 minutes (range 17-50 minutes). The duration of the interviews in phase II was on average 24 minutes (range 13-49 minutes). |
| 22. Data saturation | In phase I, we interviewed until we achieved data saturation, and for confirmation we conducted two more interviews. In phase II, we performed improvement cycles until no more considerable issues emerged. |
| 23. Transcripts returned | Transcripts were not returned to participants. |
| Domain 3: analysis and findings |  |
| Data analysis |  |
| 24. Number of data coders | EV and ES independently coded all 18 transcripts of the phase I interviews using an inductive thematic analysis. In this approach, the analysis was data driven and themes were constructed without a pre-existing frame. The researchers started by giving initial codes to relevant quotes. Subsequently, the codes were compared and when codes differed, we discussed until we reached consensus, if needed with a third person (WN). All codes were grouped into categories derived from the data through constant comparison and review. In forming the categories, the researchers regularly discussed and rearranged the quotes and codes. Atlas.ti was used for coding. The interviews of phase II were not coded, but the emerging feedback was discussed with the expert team and used to develop the next version of the tool. |
| 25. Description of the coding tree | The themes that emerged in the phase I interviews and that were viewed as important by patients were used in the tool. |
| 26. Derivation of themes | Themes were derived from the data. |
| 27. Software | We used Atlas.ti for coding. |
| 28. Participant checking | Participants were not asked to provide feedback on the findings. |
| Reporting |  |
| 29. Quotations presented | No quotations were presented |
| 30. Data and findings consistent | There was good consistency between data and findings of the phase I interviews, we used descriptions directly derived from the interviews to describe the themes in the tool. |
| 31. Clarity of major themes | The major themes of the phase I interviews are presented in the tool. The major suggestions from the phase II interviews are described in the results of this paper. |
| 32. Clarity of minor themes Is there a description of diverse cases or discussion of minor themes? | There were no minor themes. |

Reference: Tong A, Sainsbury P, Craig J. Consolidated criteria for reporting qualitative research (COREQ): a 32-item checklist for interviews and focus groups. *Int J Qual Health Care* 2007; **19**, 349–357. doi: 10.1093/intqhc/mzm042
