# Supplementary material for: The Tell me tool: The development and feasibility of a tool for person‐centred infertility care
Source: Health Expect. 2022 Feb 26;25(3):1081–93. doi: 10.1111/hex.13455 (PMC9122469; doi:10.1111/hex.13455)
Supplement: Supplementary file 2 — Supplementary Information [file HEX-25--s002.docx]

**Article Title:** The Tell me tool: the development and feasibility of a tool for person-centered infertility care.

**Supporting information 1. Interview topic guide**

1. How are you?
2. How is the treatment process going?
3. What do you think is important concerning your fertility treatment?
   - How would the care process be tailored to your needs?
   - Both organizational and human factors can be important
4. According to scientific research, these aspects were important to patients
   - Treatment success (pregnancy, obtained semen)
   - Information and explanation concerning the treatment (information about treatment options, treatment trajectory, clarity of information, possibility to ask questions)
   - Organizational aspects of care (waiting times, flexible appointments)
   - Accessibility (by phone, also outside office hours)
   - Consensus and collaboration between clinicians (seeing the same clinicians, information-exchange, same treatment policy)
   - Comfort (pain alleviation, waiting room not mixed with pregnant women, privacy, ambiance)
   - Skills of the clinicians involved in the treatment (clinicians’ attitude, medical expertise, relationship with patient)
   - Emotional support (support by a social worker or psychologist, attention for relationship)
   - Involvement in your care (of your partner, involvement in decision making, access to your medical record)
5. Which three aspects (see above) are most important to you and your partner? Do you agree or disagree on these aspects?
6. I assume you are in this trajectory to become pregnant.
   - How important is that for you?
   - What ‘price’ do you want to pay for that? How far would you go?
   - What are your treatment goals? After this treatment I want to be pregnant and …
7. We have listed five elements that are important in life: mood, physical health, daily activities (work), relationship, social. You can divide 100 points over these elements. How many points would you give to these elements and how many points for pregnancy?
8. What would be the best way to integrate such a list (question 7) in a questionnaire?
9. What would be a good way to ask patients how they cope with aspects mentioned in question 4? How would you prefer answering this question?
10. Showing examples of questionnaires / rating scales: which do you prefer?
11. Are there any other topics we did not discuss so far that we should take into account?
