# Supplementary material for: The Tell me tool: The development and feasibility of a tool for person‐centred infertility care
Source: Health Expect. 2022 Feb 26;25(3):1081–93. doi: 10.1111/hex.13455 (PMC9122469; doi:10.1111/hex.13455)
Supplement: Supplementary file 3 — Supplementary Information [file HEX-25--s001.docx]

**Article Title:** The Tell me tool: the development and feasibility of a tool for person-centered infertility care.

**Supporting information 2. Main themes derived from the semi-structured interviews**

| Categories | Overarching themes derived from semi-structured interviews |
| --- | --- |
| Treatment process | Most important  -competence of staff  -information provision  Moderate important  -attitude of/relationship with staff  -accessibility and appointments  -continuity and transition  -communication  -patient involvement and privacy  -physical comfort  Least important  -emotional support  -coordination and integration |
| Wellbeing during and after treatment | Most important  -pregnancy  -relationship  -physical health  -mental health  Least important  -Work  -finance  -social network |
