# Supplementary material for: The Tell me tool: The development and feasibility of a tool for person‐centred infertility care
Source: Health Expect. 2022 Feb 26;25(3):1081–93. doi: 10.1111/hex.13455 (PMC9122469; doi:10.1111/hex.13455)
Supplement: Supplementary file 4 — Supplementary Information [file HEX-25--s003.docx]

**Article Title:** The Tell me tool: the development and feasibility of a tool for person-centered infertility care.

**Supporting information 3. The tool used in the first improvement round (after consensus meeting)**

**What is important for your fertility treatment?**

Explanation.

Very important

Not important

Score Notes:

Becoming pregnant □□□□□□□ ____ _______________________________________________

Maintaining a good relationship with your partner □□□□□□□ ____ _______________________________________________

Staying physically healthy □□□□□□□ ____ _______________________________________________

Staying mentally healthy and happy □□□□□□□ ____ _______________________________________________

Trusting the caregivers expertise □□□□□□□ ____ _______________________________________________

Receiving enough information □□□□□□□ ____ _______________________________________________

Being able to ask questions □□□□□□□ ____ _______________________________________________

Flexible scheduling of appointments □□□□□□□ ____ _______________________________________________

Being involved in decision making □□□□□□□ ____ _______________________________________________

A pleasant attitude of/relationship with caregivers □□□□□□□ ____ _______________________________________________

_______________________________________ □□□□□□□ ____ _______________________________________________

_______________________________________ □□□□□□□ ____ _______________________________________________

What do you value in life? What values are important to you? (honesty, trust, openness, religion) _______________________________________________

______________________________________________________________________ _______________________________________________

Describe yourself in a few keywords. _______________________________________________

______________________________________________________________________ _______________________________________________
